# Network Pharmacology Combined With Metabonomics and Transcriptomics Reveals the Mechanism of *Rubus* Chingii Hu in Alleviating Nephrotoxicity Caused by Tripterygium Glycosides Tablet

**DOI:** 10.1002/fsn3.72189

**Published:** 2026-08-04

**Authors:** Cong Zhang, Zepeng Zhang, Tong Zhou, Lei Zhang

**Affiliations:** ^1^ Kunshan Rehabilitation Hospital Kunshan China; ^2^ Traditional Chinese Medicine Hospital of Kunshan Kunshan China

**Keywords:** glycerophospholipid metabolism, metabonomics, nephrotoxicity, *Rubus* chingii Hu, transcriptomics, tripterygium glycosides tablet

## Abstract

Researches on Tripterygium glycosides tablets (TGT) often overlook renal toxicity from prolonged use. *Rubus* chingii Hu (RCH) is beneficial to kidney health and has an unclear role in reducing nephrotoxicity. This study used UHPLC‐Q Exactive HFX to identify Kaempferol, Hydroxygenkwanin, and Luteolin as key active components of RCH. Network pharmacology revealed RCH could protect the kidneys by modulating TNF, IL‐17, NF‐kappa B, and JAK–STAT pathways through targets such as BCL2, CASP3, TNF, SIRT1, EGFR, and MMP9. Biochemical and pathological studies show that TGT induces kidney injury in rats, while RCH improves kidney function and reduces inflammation and oxidative stress. Western blot confirmed that RCH could regulate the expression of TNF‐α, cleaved caspase‐3, and SIRT1 proteins. Transcriptomics and metabonomics reveal significant enrichment in metabolic pathways like glycerophospholipid metabolism and inflammation signaling pathways such as PPAR and JAK–STAT. After RCH treatment, most differential genes remain stable, but all 20 key metabolites are restored. Further analysis indicates that steroid hormone biosynthesis and the PPAR signaling pathway are vital for RCH's protective effects, as it regulates CYP1B1, CYP4A1, CYP4A3, EHHADH, HMGCS2, and PPARG genes, alleviating TGT‐induced nephrotoxicity.

## Graphical abstract

Flow chart of RCH to improve TGT‐induced nephrotoxicity.

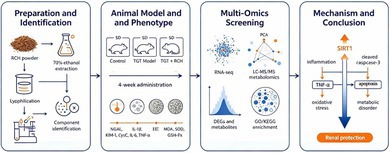

## 1 Introduction

Kidney is an important organ of the human body, which can produce urine, regulate acid–base balance, maintain electrolyte balance, and remove wastes and toxic substances from the body (Yadav et al. 2024). Its normal structure and function play a crucial role in maintaining dynamic balance in the body (Liu et al. 2015). Many factors such as pathology and external stress may have adverse effects on the kidney and further interfere with the fluid balance in the body (Wang et al. 2024), and different degrees of kidney function damage may develop into end‐stage renal disease. In terms of mechanism, kidney damage may be due to hemodynamic effects, renal tubular or glomerular toxicity, and interstitial nephritis (Khan et al. 2017). At present, nephrotoxicity is mainly related to the use of drugs (Kwiatkowska et al. 2021).

*Tripterygium wilfordii* is a famous traditional Chinese medicine in China. Because of its remarkable anti‐inflammatory and immunosuppressive effects, it is widely used to treat rheumatoid arthritis and systemic lupus erythematosus (Song et al. 2020). Tripterygium glycosides is an effective component of saponins extracted from the root bark of *Tripterygium wilfordii* Hook F, diterpenoids such as triptolide and triptonide, triterpenoids such as wilforlide A and celastrol, and alkaloids such as wilforine and wilforgine are its main effective and toxic components (Chen et al. 2022; Liu et al. 2021; Dong et al. 2019). Tripterygium glycosides tablet (TGT) with these as the main components is currently the most widely used Chinese medicine preparation in clinic and has the best curative effect on autoimmune and inflammatory diseases (Wang et al. 2017). However, there were more and more reports of clinical adverse reactions of TGTs (Wang et al. 2022). At present, many studies on the toxicity of TGT focused on the liver and reproductive toxicity (Qian et al. 2022; Dai et al. 2022), there were few and in‐depth studies on nephrotoxicity, but there was evidence that long‐term or high‐dose use of TGT will lead to kidney injury and even death (Wang, Wang, et al. 2019; Liu et al. 2019), clinical data showed that the incidence of *Tripterygium wilfordii* preparation‐related nephrotoxicity is 5.81% (Feng et al. 2019). These toxic and side effects limit its wider clinical application. Therefore, it is necessary to further reveal the nephrotoxicity mechanism that is not yet fully understood by TGT and develop drugs that effectively slow or treat nephrotoxicity.

Specifically, the clinical nephrotoxicity of TGT is mainly manifested as inflammatory cell infiltration, renal tubular atrophy or edema, and renal pelvis dilatation (Luo et al. 2018). Current studies have shown that nephrotoxicity caused by TGT is related to oxidative stress damage, activation of apoptosis, and dysregulation of drug transport. This involves various mechanisms, including the decrease in the expression of renal antioxidant factors glutathione peroxidase (GSH Px), superoxide dismutase (SOD), catalase (CAT) and anti‐inflammatory factors IL‐10 and the increase in the level of pro‐inflammatory factor TNF‐α, as well as tubular apoptosis caused by up‐regulation of the ratio of Bax to Bcl‐2, and the Organi cation transporter 2 (OCT2) transports excessive toxic components to renal tubular cells, resulting in the inability of drug‐transporting efflux protein (ABCB1, ABCC2) to expel excess components in time, exacerbating toxicity (Wang, Li, et al. 2019; Allard et al. 2013; Shen et al. 2019). In addition, metabolic disorders are also thought to be related to the progression of nephrotoxicity and interventions aimed at ensuring its repair (Niemann and Serkova 2007). Unfortunately, adequate doses and courses of treatment are necessary to ensure the efficacy of the drug, but current improvements in nephrotoxicity caused by TGT are limited, which means that treatment is abandoned or its attenuation needs to be promoted. Therefore, it is essential to develop a compatible detoxification strategy of traditional Chinese medicine based on component‐target‐effector interactions. This strategy achieves compatible detoxification by blocking drug‐mediated damage and promoting damage repair. Specific mechanisms include component interactions, target interactions and/or effector interactions (Bai et al. 2025). This method of synergistic attenuation has been proved to be feasible (Zhang et al. 2022). However, no effective measures have been taken to treat TGT‐induced kidney damage.

At the same time, due to the complex interactions between the various components of RCH, further research is needed to understand its active components and their mechanisms of action. At present, multi‐omic research based on sequencing technology and high‐resolution mass spectrometry technology is developing rapidly, providing an effective way to discover potential compounds and understand their overall biological mechanisms and reveal regulatory networks in specific biological processes (Wu et al. 2021). In recent years, transcriptomics has been widely used to reveal the mechanism of traditional Chinese medicine regulating the occurrence and progress of diseases (Xu et al. 2025), while metabolomics can be used to identify the dynamic changes of key endogenous small molecule metabolites and complex metabolic pathways in biological samples (Lin, Tian, et al. 2024; Fernandes Silva and Laakso 2025). Based on this, this study conducted a joint analysis of kidney metabolites and genes before and after RCH intervention, aiming to accurately locate the metabolic pathway from gene to disease (Yin et al. 2022), thereby exploring the effect and potential mechanism of RCH in improving TGT‐induced nephrotoxicity and providing a strategy for enhancing efficiency and reducing toxicity for future treatment of TGT.

## 2 Materials and Methods

### 2.1 Preparation of RCH Extract

Three hundred grams of RCH (Suzhou Tianling Chinese Herbal Pieces Co. Ltd., Jiangsu, China) powder was dissolved in 3 L of 70% ethanol, allowed to stand for 1 h and then refluxed and extracted twice, each time for 1 h (Sunil et al. 2023). The filtrates were combined and concentrated to a concentration of about 1 g/mL by rotary evaporator (N‐1300D‐WB, EYELA, Japan), and then freeze‐dried and preserved. According to the dry weight of freeze‐dried powder, the yield of the extract was 11.1%.

### 2.2 RCH Component Identification

The RCH extract was created with 70% methanol by Ultrasonic Cleaner (KQ5200DM, Kunshan Ultrasonic Instrument Co. Ltd. China), centrifuged (12,000 r/min, 15 min) with high speed centrifuge (5430R, Eppendorf, Germany), and filtered with 0.22 μm membrane to obtain a clear supernatant. Subsequently, the components of RCH were analyzed by ultra high performance liquid chromatography (Vanquish, UPLC, Thermo, USA) and quadrupole exactive hybrid mass spectrometer (Q Exactive HFX, Thermo, USA). In this process, the mobile phase consists of 0.1% formic acid‐water solution (A) and 0.1% formic acid‐acetonitrile (B). Using elution gradient method, the initial 0% B lasts for 1 min, then it rises to 95% B at 12.0 min for 1 min, and then falls to 0% B within 0.1 min and lasts until 17.0 min. The flow rate was 0.3 mL/min, the column temperature was kept at 40°C, and the injection volume was 2 uL. Then, electrospray ionization‐MS analysis was performed. The ion source temperature was 320°C, the positive ion mode voltage was 3.0 kV, the negative ion mode voltage was 2.8 kV, the sheath gas was 40 arb, and the auxiliary gas was 10 arb. Data was collected in full scanning mode with a mass scan range of m/z 70–1050 Da. Using nitrogen as the medium, specific multiple reaction monitoring (MRM) ion pairs were monitored during each cycle (Chen et al. 2024). MS data were processed by Progenesis QI (Waters Corporation, Milford, USA) software and compared with the proprietary secondary spectrum database of Sanshu Biotechnology Co. Ltd. The matching degree of secondary mass spectrometry (MS2) was evaluated using fragment ion spectrum similarity score, and the total score was 1. The higher the score, the more reliable the identification result is. The reliability threshold set in the experiment is score ≥ 0.7 (Neto and Raftery 2021).

### 2.3 Network Pharmacological Analysis

Using the Traditional Chinese Medicine Systems Pharmacology Database and Analysis Platform (TCMSP, https://tcmspw.com/tcmsp.php), the components meeting the standards of oral bioavailability (OB) ≥ 30% and drug‐like activity (DL) ≥ 0.18 were screened out (Wu et al. 2022). In addition, the corresponding targets (Probability* > 0) were retrieved using the swisstargetprediction database (STP, https://swisstargetprediction.ch/). Using “Nephrotoxicity” and “Renal Toxicity” as keywords, the disease‐related genes were obtained from Online Mendelian Inheritance in Man (OMIM, https://www.omim.org/), GeneCards (http://www.genecards.org) and Drugbank (https://go.drug bank.com/) databases. The intersection targets of nephrotoxicity‐related genes and active targets of RCH were obtained through Venny 2.1.0 (https://bioinfogp.cnb.csic.es/tools/venny/index.html). The intersection targets were imported into the STRING database (https://string‐db.org/) and the protein–protein interaction (PPI) network was visualized using Cytoscape 3.7.2 (https://cytoscape.org/) software. Referring to the DAVID database (https://davidbioinformatics.nih.gov/), the intersection targets were subjected to gene ontology (GO) and Kyoto Encyclopedia of Genes and Genomes (KEGG) pathway enrichment analysis (Dou et al. 2024).

### 2.4 Animals and Experimental Design

Eighteen SPF male Sprague–Dawley (SD) rats (220 ± 20 g) were purchased from Shanghai Slick Experimental Co. Ltd. (License Number: SCXK (HU) 2022‐0004). During the experiment, rats were kept in a controlled environment with a temperature of 22°C ± 2°C, a humidity of 50% ± 5%, and a 12‐h light/dark cycle. All experiments in this study were approved by the Institutional Ethics Committee of Zhejiang Huitong Evaluation Technology (Group) Co. Ltd. (Approval Number: HTDW‐202405002) and ensured full compliance with the standards of the established “Guidelines for the Management and Care of Laboratory Animals”.

After adapting to the environment for one week, the rats were randomly divided into 3 groups (*n* = 6/group), including the control group (Con), TGT group (Mod), and TGT + RCH group (RCH). Prepared RCH solution in distilled water. The model group was given 135 mg kg^−1^ (15 times the clinical equivalent dose) of TGT (Qianjin Xieli Pharmaceutical Company Ltd., Hunan, China) by gavage (Fu et al. 2023). The RCH group was given equal doses of TGTs by gavage in the morning and 400 mg kg^−1^ (3 times the clinical equivalent dose) of RCH solution by gavage in the afternoon. At the same time, rats in the control group and model group were given equal doses of saline daily. The above treatments were given once daily for 4 weeks. After the last dose, urine samples were collected from rats fasted for 12 h. All rats were anesthetized with pentobarbital, blood was taken by abdominal aortic puncture, and centrifuged at 3000 rpm for 10 min at 4°C. Serum was stored with kidney tissue at −80°C until analysis.

### 2.5 Assessment of Kidney Function and Histopathology

Urine was centrifuged (1000 r/min, 15 min, 4°C) to collect supernatant, and the contents of neutrophil gelatinase‐associated lipocalin (NGAL) and kidney injury molecule‐1 (KIM‐1) in neutrophils were detected according to the instructions of enzyme‐linked immunosorbent assay (ELISA) kit (Nanjing Boyan Bio, Jiangsu, China) (Weber et al. 2026). The centrifuged rat serum was taken out of the refrigerator at −80°C, and the serum cystatin C (CysC) (Beyotime Biotechnology, Shanghai, China) level was detected by COBAS 6000 automatic biochemical analyzer (Roche, Switzerland) (Zhu et al. 2021). After the kidney was fixed, it was dehydrated and embedded, made into 5 μm slices and stained with hematoxylin–eosin (HE), observed under a microscope (NIKON DS‐U3, Japan) and collected images at 20 times magnification according to the principle of maximizing commonality and core features (Huang, Zhang, et al. 2025).

### 2.6 Detection of Inflammatory, Oxidative Stress Indexes and Related Proteins in Renal Tissue

Take a kidney sample, remove blood stains, add human cold physiological saline at a ratio of 1:9 for ice bath homogenization, centrifuge the tissue homogenate at 15000 r/min for 10 min, and suck the supernatant (Borges et al. 2025). BCA Protein Assay Kit (Beyotime Biotechnology, Shanghai, China) was used to detect the total protein content in each homogenate sample. According to the operation instructions of the kit (Nanjing Jiancheng Institute of Bioengineering, Jiangsu, China), the contents of Interleukin‐1β (IL‐1β), Interleukin‐6 (IL‐6), and Tumor necrosis factor‐alpha (TNF‐α) in the homogenate were detected by ELISA, malondialdehyde (MDA) by thiobarbituric acid method, and SOD and GSH‐Px by microplate method (An et al. 2021).

In addition, Western blot was performed to detect relevant proteins in kidney tissue. Briefly, RIPA lysis buffer (Jingcai Biotech, Xi'an, China) was added to kidney tissue, which was then homogenized. Total protein concentration was determined using a BCA assay kit (Beyotime Biotech, Shanghai, China). The protein sample was mixed thoroughly with 5× protein loading buffer (ZOMANBIO, Beijing, China) at a ratio of 4:1 (v/v). Proteins were separated by electrophoresis on SDS‐PAGE stacking gel (Jingcai Biotech, Xi'an, China) and transferred to PVDF membrane, blocked with 5% skim milk at room temperature for 2 h, and incubated at 4°C with TNF‐α (1:1500), Cleaved caspase‐3 (1:1000), SIRT1 (1:1500), and GAPDH (1:2000) primary antibodies (all from Sanying Biotech, Wuhan, China) overnight, incubated with HRP secondary antibody (1:5000) for 1 h, and then combined with ECL luminescent solution (Pierce ECL Plus, Thermo Scientific) to measure the gray values of the protein bands.

### 2.7 Transcriptome Sequencing and Data Analysis of Kidney Tissue

Transcriptomic sequencing was performed on kidney tissues of rats from the Con, Mod and RCH group (*n* = 3) by Sanshu Biotechnology Co. Ltd. (Nantong, China). In short, total RNA from the samples was extracted with TRIzol reagent, and after concentration determination and two rounds of purification, the total RNA was extracted using the Nova‐seq6000 platform (Illumina Inc., San Diego, CA, USA) sequencing RNA libraries (Arance et al. 2021). Then, Spliced Transcripts Alignment to a Reference (STAR) software was used to perform comparisons with the reference genome. Finally, DESeq2 (https://bioconductor.org/packages/release/bioc/html/DESeq2.html) software was used to screen for differentially expressed genes (DEGs) between the two groups. Genes with padj value < 0.05 and |log2(foldchange)| (FC) > 1.5 were selected as differentially expressed genes (Li et al. 2025). Subsequently, the criterion of limiting significant enrichment is *p* < 0.05, and the GO items with significant enrichment of differential genes were found, and the main biological functions performed by differential genes were determined, and the GO and KEGG pathways of differential genes were enriched and analyzed by Eggnog‐mapper (https://eggnogdb.org/) software (Hernández‐Plaza et al. 2026).

### 2.8 Untargeted Metabolomics of Kidney Tissue

#### 2.8.1 Sample Processing

Kidney tissue samples (100 mg) of rats in the Con, Mod, and RCH group (*n* = 3) were transferred to centrifuge tubes, 0.2 mL of pre‐cooled water was added, mixed by Vortex‐Genie 2 (Scientific Industries, USA) for 60 s (Chen et al. 2026); then 0.8 mL of pre‐cooled extraction solvent (methanol/acetonitrile, 1:1, v/v) was added, vortexed, and mixed again for 60 s, extracted by low‐temperature ultrasound for 30 min, and the protein was precipitated at 20°C for 1 h, and centrifuged at 12,000 rpm for 10 min. The obtained precipitate was concentrated in vacuum, then resuspended with 200 μL of 30% acetonitrile‐water solution, and fully vortexed. Finally, the sample was ultracentrifuged at 4°C at 14,000 rpm for 15 min at low temperature (Zhang et al. 2021). The final supernatant was collected for LC–MS/MS analysis.

#### 2.8.2 UHPLC‐Q Exactive HFX Analysis

The analysis of UHPLC–MS/MS was carried out by combining Vanquish UPLC system (ThermoFisher Scientific, Germany) with Orbitrap Q Exactive HFX analysis (ThermoFisher Scientific, Germany). Specifically, the sample was injected by Waters HSS T3 reversed‐phase column (100 × 2.1 mm, 1.8 μm), the column temperature was 40°C, the flow rate was 0.3 mL/min, the injection volume was 2 μL, and the linear gradient elution was used for 8 min. The mobile phase consists of phase A (0.1% formic acid aqueous solution) and phase B (0.1% formic acid acetonitrile solution) (Wen et al. 2022). The gradient elution program was configured as follows: isocratic elution (A:B = 100:0) was performed from 0 to 1 min, followed by linear gradient elution from 1 to 4 min to A:B = 40:60, then elution to A:B = 5:95 at 6.5 min, reaching A:B = 100:0 at 6.6 min and maintained for 8 min. All samples were analyzed in turn by a temperature‐controlled autosampler set at 4°C.

Mass spectrometry analysis was performed in positive and negative ion mode. The auxiliary gas and sheath gas were both nitrogen, and the flow rates are 10 arb and 40 arb respectively. The ion ejection voltage was 3 kV in positive ion mode and 2.8 kV in negative ion mode. The ion source temperature and auxiliary gas heating temperature were 320°C and 350°C respectively (Wang et al. 2021). The fragment voltage was set to 50 V, and positive/negative ions were scanned in Full MS/ddMS2 mode, with scanning mass ratio range from m/z 70 to 1050 Da.

#### 2.8.3 Data Processing and Multivariate Analysis

The raw data files were processed using Progenesis QI software, which mainly included peak identification, peak matching and retention time correction. The aligned data matrix was normalized using total area normalization. SIMCA14.1 software was used to perform multivariate analysis, including principal component analysis (PCA) and orthogonal projection to latent structure discriminant analysis (OPLS‐DA). The OPLS‐DA model was verified by 200 substitution tests and assessed by Q2 values (Qin et al. 2023). Combining the score graph of OPLS‐DA with the variable importance projection (VIP) graph, metabolites with Fold Change (FC) greater than 1.5 or less than 0.667 were considered potential biomarkers when VIP values were greater than 1 and *p* values < 0.05 (Ban et al. 2021). Metabolites were subsequently annotated and enriched via the KEGG database (https://www.genome.jp/kegg/pathway.html), the human metabolite database HMDB (https://hmdb.ca) and Metaboanalyst (www.metaboanalyst.ca) (Wang, Shan, et al. 2023).

### 2.9 RT‐PCR Verification of Core Genes

The mRNA expression of core DEGs was confirmed by RT‐PCR. The remaining samples of total RNA extracted by transcriptome sequencing were reverse transcribed into cDNA templates and PCR amplified according to the instructions. According to the Ct value of each group, β‐actin was selected as the internal reference gene, and the relative expression of mRNA in each group of samples was calculated by the 2^−ΔΔCt^ method. Primer sequences are given in Table S1 (Supporting Information).

### 2.10 Statistical Analysis

The experimental data were processed with SPSS26.0 software and expressed as mean ± standard deviation (SD). One‐way ANOVA was used for comparisons among groups, and the LSD test was used for comparison between pairs. *p* < 0.05 indicates that the difference is statistically significant.

## 3 Results and Discussion

### 3.1 Identification Results of the Main Components of RCH

**FIGURE 1:**
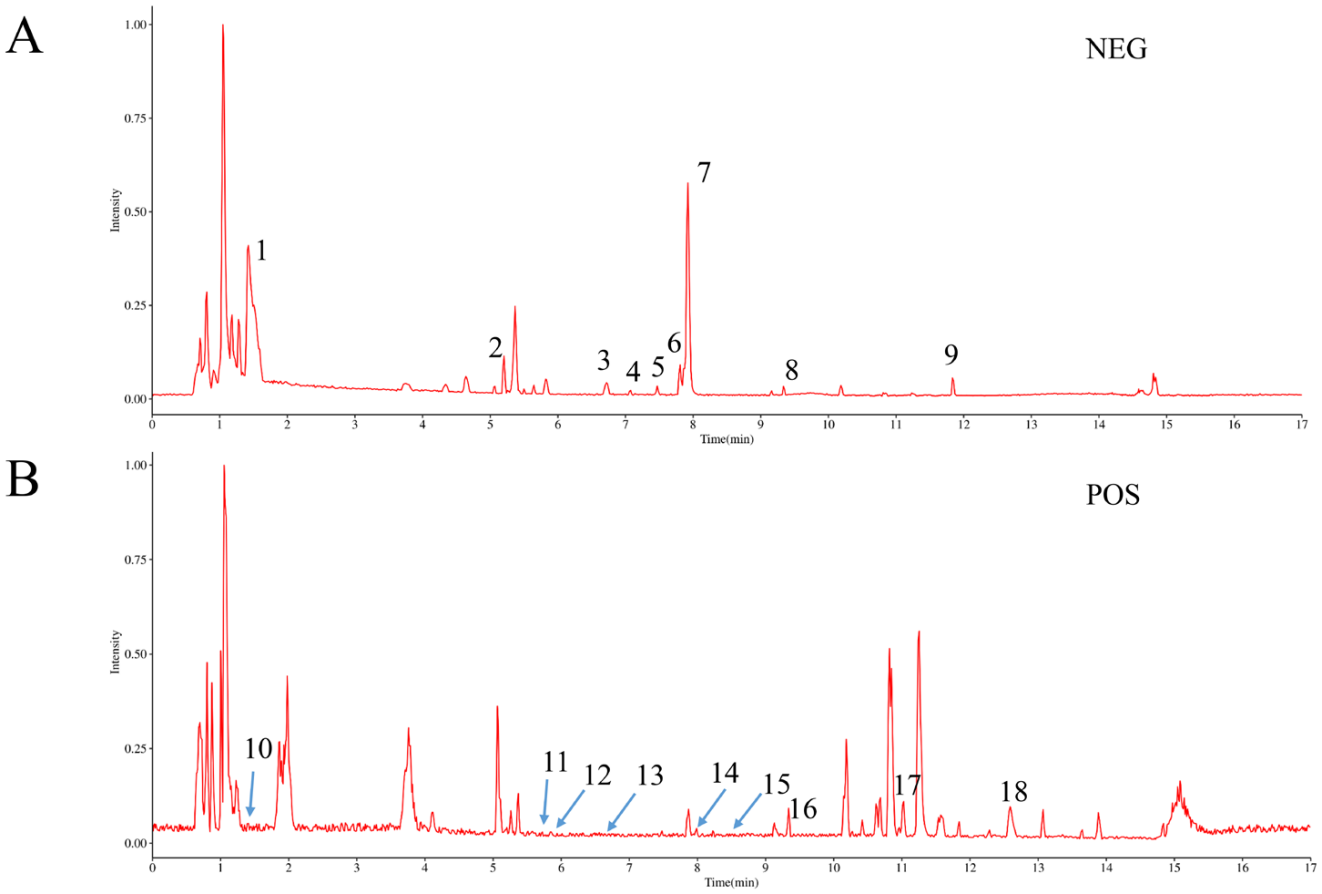
Total ion current (TIC) chromatograms of RCH obtained by UHPLC‐Q Exactive HFX: (A) Negative ion mode (NEG); (B) Positive ion mode (POS).

Several studies have shown that the unique multi‐target, multi‐component and multi‐channel advantages of traditional Chinese medicine and compound preparations show their potential as an auxiliary means of traditional treatment strategies for TGT toxicity (Zhang et al. 2023; Han et al. 2023). In previous studies, the water‐alcohol extract of RCH has been shown to protect the kidney injury of methotrexate rats (Khoshnoud et al. 2017). Nevertheless, the identification of the active ingredient is a prerequisite for elucidating the efficacy and therapeutic mechanism of RCH in addressing TGT‐induced kidney injury. In this study, we used UHPLC‐Q Exactive HFX technology to characterize the extract of RCH, thereby obtaining relatively accurate active compounds. Based on the total ion patterns generated by RCH in positive and negative modes, the peak information was matched with the self‐built library, and the existing chemical components were preliminarily confirmed. The mass extraction limit of 5 ppm was used for data analysis (Yi et al. 2020), and 18 main active components of RCH were finally determined according to the OB and DL values (Figure 1 and Table 1). Overall, flavonoids accounted for the majority (10, 55.6%), with others including isoflavones (2, 11.1%) as well as flavonoid glycosides, glycosides, polyphenols, coumarins, diterpenes, and triterpenoids. This shows that 70% ethanol extract can show the highest content of polyphenols, flavonoids and their derivatives, which is because 70% ethanol induces the most significant structural changes in cell walls, and these compounds have anti‐inflammatory and antioxidant potential (Cheng et al. 2025).

**TABLE 1:** The active ingredient of RCH identified by UHPLC‐Q Exactive HFX.

No.	Ingredient	Mode	Formula	RT/min	OB (%)	DL
1	Aucubin	Neg	C_15_H_22_O_9_	1.458	35.56	0.33
2	Herbacetin	Neg	C_15_H_10_O_7_	5.253	36.07	0.27
3	Luteolin	Neg	C_15_H_10_O_6_	6.698	36.16	0.25
4	Baicalin methyl ester	Neg	C_22_H_20_O_11_	7.157	40.12	0.75
5	Glycyrrhiza flavonol A	Neg	C_20_H_18_O_7_	7.529	41.28	0.60
6	Hesperetin	Neg	C_16_H_14_O_6_	7.751	70.31	0.27
7	Hydroxygenkwanin	Neg	C_16_H_12_O_6_	8.053	36.47	0.27
8	Medicarpin	Neg	C_16_H_14_O_4_	9.378	49.22	0.34
9	Poricoic acid B	Neg	C_30_H_44_O_5_	11.831	30.52	0.75
10	Ellagic acid	Pos	C_14_H_6_O_8_	1.321	43.06	0.43
11	Quercetin	Pos	C_15_H_10_O_7_	5.586	46.43	0.28
12	Kaempferol	Pos	C_15_H_10_O_6_	5.884	41.88	0.24
13	Glabrene	Pos	C_20_H_18_O_4_	6.655	46.27	0.44
14	Isorhamnetin	Pos	C_16_H_12_O_7_	7.960	49.60	0.31
15	Formononetin	Pos	C_16_H_12_O_4_	8.570	69.67	0.21
16	Wogonin	Pos	C_16_H_12_O_5_	9.270	30.68	0.23
17	Isoimperatorin	Pos	C_16_H_14_O_4_	10.990	45.46	0.23
18	Tanshinone IIA	Pos	C_19_H_18_O_3_	12.659	49.89	0.40

### 3.2 Network Pharmacological Analysis

**FIGURE 2:**
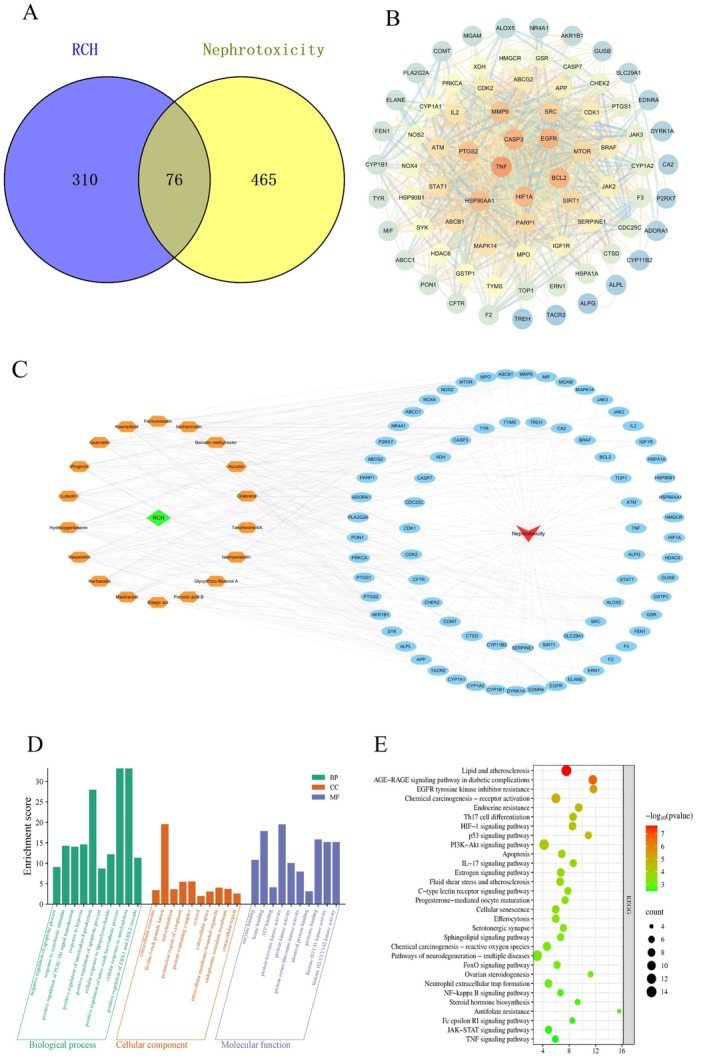
Results of network pharmacology. (A) The targets of RCH to improve nephrotoxicity (B) PPI network (C) “Drug‐Component‐Target‐Disease” network (D) GO enrichment (E) KEGG enrichment.

Network pharmacology was used to identify multiple components and targets of RCH and analyze their relationship to TGT‐related nephrotoxicity targets. Based on the RCH active components determined above, 386 corresponding targets were screened, and 76 overlapping targets were obtained by crossing with 541 nephrotoxicity targets (Figure 2A). From this, we built a PPI network with medium reliability (0.4) (Figure 2B). Using Maximal Clique Centrality (MCC) algorithm of cytoHubba plug‐in, PPI network was analyzed to locate key genes quickly. The results (Table 2) showed that 12 targets including BCL2, CASP3, TNF, EGFR, MMP9, MTOR, and SIRT1 were identified as hub targets. Analysis of “drug‐component‐target‐disease” network (Figure 2C) suggested that Kaempferol, Hydroxygenkwanin, and Luteolin with the greatest degree of freedom may be the core active components of RCH in treating nephrotoxicity.

**TABLE 2:** Analysis results of core target in PPI network.

Rank	Target	Score (×10^11^)	Rank	Target	Score (×10^11^)
1	BCL2	5.84	7	HIF1A	5.83
2	CASP3	5.84	8	HSP90AA1	5.83
3	TNF	5.84	9	STAT1	5.76
4	EGFR	5.84	10	PARP1	5.62
5	MMP9	5.83	11	SIRT1	5.43
6	MTOR	5.83	12	SRC	4.90

GO analysis indicates that genes were mainly involved in biological processes related to response to xenobiotic stimulus, response to hypoxia, the regulation of the apoptosis process, and positive regulation of PI3K/Akt signal transduction, and were localized in mitochondrion, perinuclear region of cytoplasm, and cytosol region where they were associated with molecular functions such as enzyme binding, ATP binding, and protein kinase activity (Figure 2D). KEGG enrichment analysis showed that the target gene expression was mainly related to Lipid and atherosclerosis, AGE‐RAGE signaling pathway in diabetic complications, Endocrine resistance, Apoptosis, HIF‐1, IL‐17, PI3K‐Akt, TNF, NF‐κB, and JAK–STAT signaling pathway, except for cancer and other disease‐related pathways (Figure 2E). These results suggest that RCH may protect kidney by regulating inflammatory response and oxidative stress through these signaling pathways.

Many studies have confirmed that Kaempferol exerts kidney protection through multiple pathways, which can regulate key pathways such as nuclear factor κB (NF‐κB), mitogen‐activated protein kinase (MAPK), and nucleotide‐binding oligomerization domain‐like receptor protein 3 (NLRP3) inflammatome. It also activates the nuclear factor E2‐related factor 2 (Nrf2)/heme oxygenase‐1 (HO‐1) antioxidant axis, thereby significantly improving kidney damage caused by drug side effects and heavy metals (Shi et al. 2025). This protective effect is reflected in the fact that Kaempferol can alleviate drug‐induced deterioration of kidney function, tubular cell exfoliation and necrosis, and tubular vacuolation (Zhang et al. 2025). Hydroxygenkwanin (HGK) has significant anti‐inflammatory, anti‐proliferation and anti‐migration effects (Chen et al. 2025), and can protect PC12 cells from oxidative damage caused by excessive hydrogen peroxide loading, showing the ability to up‐regulate a series of endogenous antioxidant proteins (Osama et al. 2024). Luteolin is a bioactive flavonoid with kidney protection, anti‐inflammatory, anti‐oxidation and anti‐apoptosis effects. It has been found that lipopolysaccharide can cause rapid kidney damage in mice, with the increase of BUN, Scr, Kim‐1and CysC, renal tubular necrosis and increased oxidative stress. Luteolin pretreatment can obviously alleviate this kidney damage and improve kidney function (Xin et al. 2016). These findings suggest that RCH can effectively reduce the expression level of key genes in relevant signaling pathways through the combination of active components such as Kaempferol, Hydroxygenkwanin and Luteolin to reduce oxidative stress and inflammatory responses, which may contribute to its protective effect on TGT‐induced kidney damage.

### 3.3 Protective Effect of RCH on TGT‐Induced Kidney Injury in Rats

**FIGURE 3:**
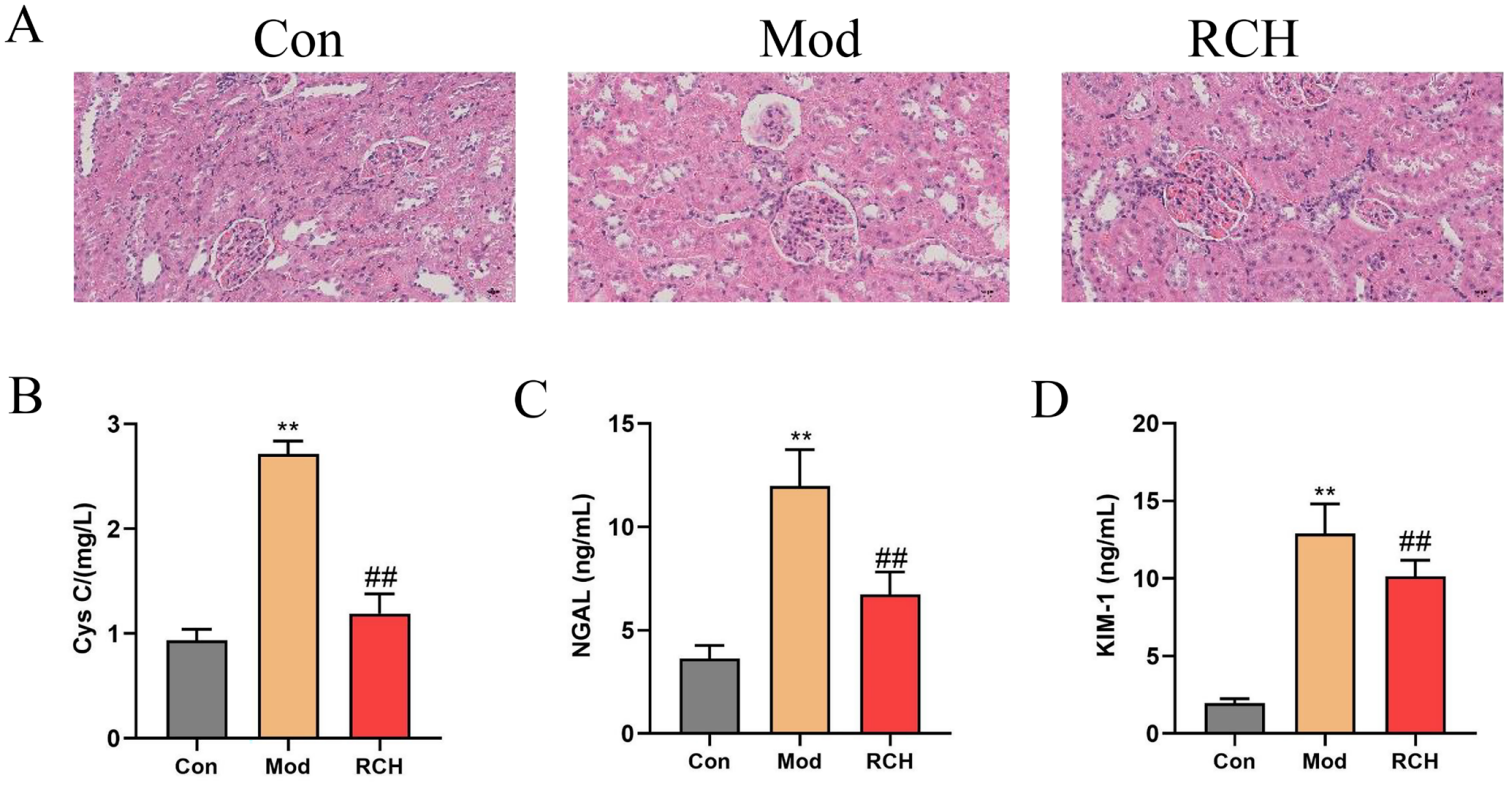
HE staining (A); CysC level (B); NGAL level (C); KIM‐1 level (D). Compared with the control group, **p < 0.01; Compared with the model (TGT) group, ^##^ *p* < 0.01.

Kidney plays a major role in maintaining homeostasis and detoxification in vivo. Due to the filtered toxin needing to be reabsorbed into the lumen, the kidney is exposed to a larger proportion of drugs and toxins than other organs, so drug‐induced kidney injury is a serious problem in clinical practice (Hosohata 2016). In order to improve the curative effect of therapeutic drugs, improving the nephrotoxicity of drugs has always been the focus of attention. Thus, in order to explore the pharmacological effect of RCH on TGT‐induced nephrotoxicity, we designed a high‐dose (135 mg kg^−1^) TGT‐induced animal study. HE staining results showed that after being given TGT for 28 consecutive days, rats in the Mod group developed pathological injuries such as renal tubular epithelial cell shedding, vacuolar degeneration, renal tubular lumen expansion, and inflammatory cell infiltration. This is consistent with the renal pathological damage caused by existing *Tripterygium wilfordii* preparations (Zhang et al. 2018). After RCH intervention, although the TGT‐induced kidney tissue structure of rats did not completely recover to normal, the degree of pathological damage was significantly reduced (Figure 3A).

Studies had shown that Kim‐1, CysC and NGAL were significantly correlated with conventional biomarkers reflecting kidney function, NGAL is significantly positively correlated with an estimated glomerular filtration rate (eGFR), KIM‐1 is positively correlated with urinary protein excretion, and KIM‐1 and NGAL concentrations can predict AKI after the use of potentially nephrotoxic drugs (Sridharan et al. 2021; Varatharajan et al. 2024). To more comprehensively assess kidney damage, NGAL and KIM‐1 levels in urine and CysC level in serum were measured (Figure 3B–D). The results revealed that TGT significantly increased the levels of CysC and NGAL, KIM‐1 in rats (*p* < 0.01). Compared with the Mod group, RCH intervention significantly reduced CysC, NGAL and KIM‐1 levels (*p* < 0.01). The above results suggest that rats given TGT have damaged kidneys and RCH can restore kidney function.

**FIGURE 4:**
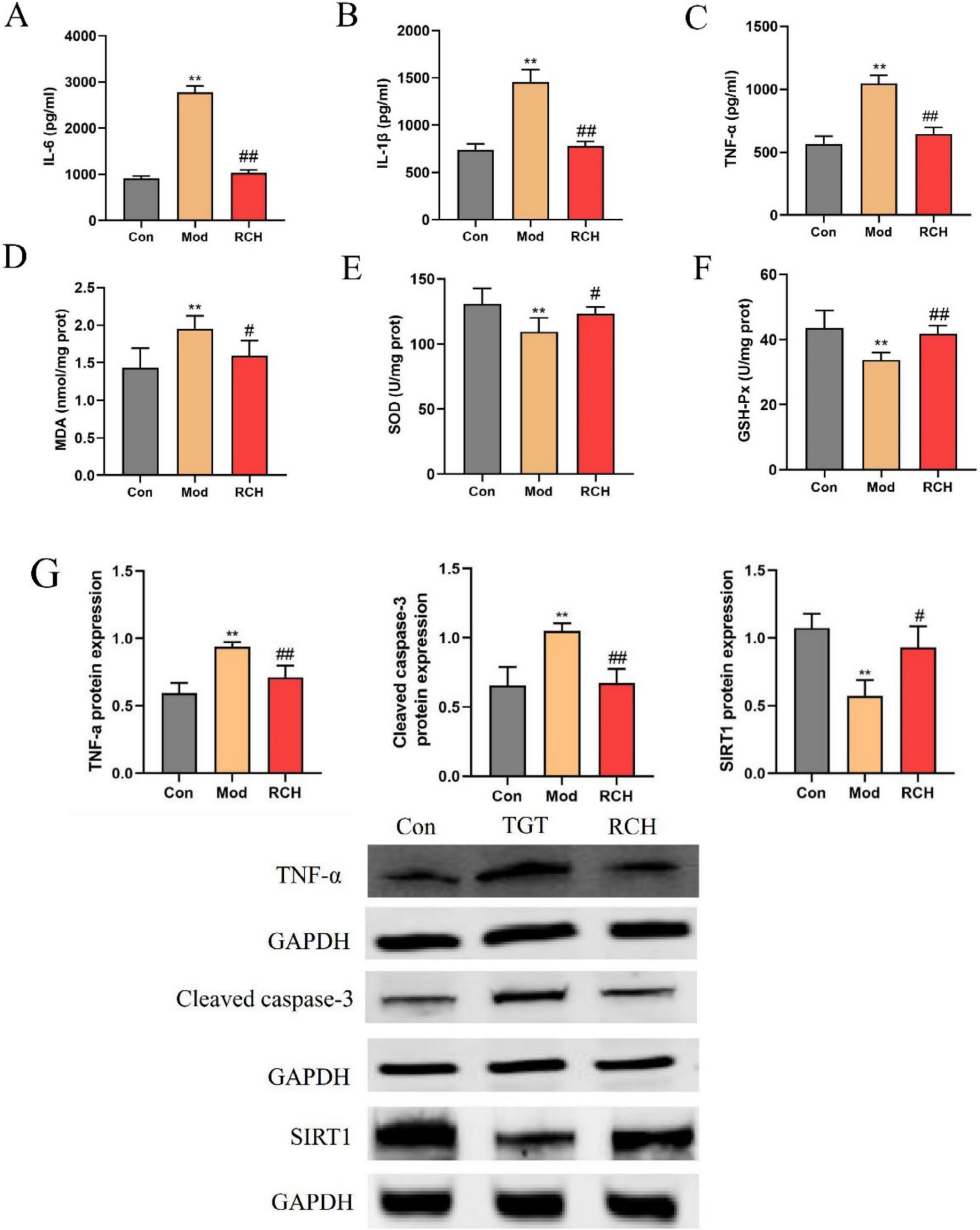
The improving effect and mechanism of RCH on TGT‐induced kidney injury (A) IL‐6 level; (B) IL‐1β level; (C) TNF‐α level; (D) MDA level; (E) SOD level; (F) GSH‐Px level. (G) Relative expression of TNF‐α, Cleaved caspase‐3 and SIRT1 proteins. Compared with the control group, **p < 0.01; Compared with the model (TGT) group, ^#^ *p* < 0.05 or ^##^ *p* < 0.01.

In addition, oxidative stress promotes drug‐induced kidney damage by activating the inflammatory response through the release of pro‐inflammatory cytokines and the accumulation of inflammatory cells in tissues, which was confirmed in this study. As shown in Figure 4A–F, compared with the Con group, the levels of IL‐6, IL‐1β, TNF‐α and MDA in kidney tissue of the Mod group increased significantly (*p* < 0.01), while the activities of SOD and GSH‐Px decreased significantly (*p* < 0.01). Compared with the Mod group, the levels of IL‐1β, IL‐6, TNF‐α and MDA in kidney tissue decreased significantly after RCH intervention (*p* < 0.01 or 0.05), while the activities of SOD and GSH‐Px increased significantly (*p* < 0.01 or 0.05). These results indicate that RCH can effectively improve the imbalance of oxidative stress and inflammation levels.

From the perspective of regulatory mechanism, as shown in Figure 4G, compared with the control group, the expressions of TNF‐a and Cleaved caspase‐3 protein in the kidney tissue of rats in the model group were significantly increased (*p* < 0.01), and the expression of SIRT1 protein was significantly decreased (*p* < 0.01). Compared with the model group, TNF‐a and Cleaved caspase‐3 proteins in the kidney tissue of rats in the RCH group were significantly down‐regulated (*p* < 0.01), and SIRT1 protein expression was significantly up‐regulated (*p* < 0.05). In recent years, studies have found that Silent information regulator 2‐related enzyme 1 (SIRT1) can play a strong renal protective role in AKI induced by different triggers such as ischemia–reperfusion injury, nephrotoxic drugs, sepsis, etc. (Wu et al. 2025; Hu et al. 2024; Tang et al. 2024). Among the active compounds identified in this study, the natural activators of SIRT1, ellagic acid and formononetin, have been proven to have antioxidant, anti‐apoptotic, and anti‐inflammatory functions, and have a protective effect on kidney disease (Naghibi et al. 2023; Singh et al. 2026). TNF‐a is a potent pro‐inflammatory cytokine and an important mediator of inflammatory tissue damage. Many experimental studies and clinical observations support the role of TNF in the pathogenesis of acute and chronic kidney disease, involving cell proliferation, cell death, and induction of inflammation and immune regulation (Vielhauer and Mayadas 2007; Al‐Lamki and Mayadas 2015). Apoptosis is a form of programmed cell death, and caspase‐3, as a key protease, is involved in the execution of apoptotic cell death and the regulation of inflammation (Kumar et al. 2022). Especially when it is cleaved into cleaved caspase‐3 (the activated form of caspase‐3), it becomes the most important terminal shearing enzyme in the process of apoptosis, directly participating in the core link of the cell killing mechanism (Dho et al. 2025). This study demonstrates the potential of RCH to improve TGT‐induced nephrotoxicity through anti‐inflammation, anti‐oxidative stress, and anti‐apoptosis.

### 3.4 Transcriptomic Analysis of Kidney Tissue

**FIGURE 5:**
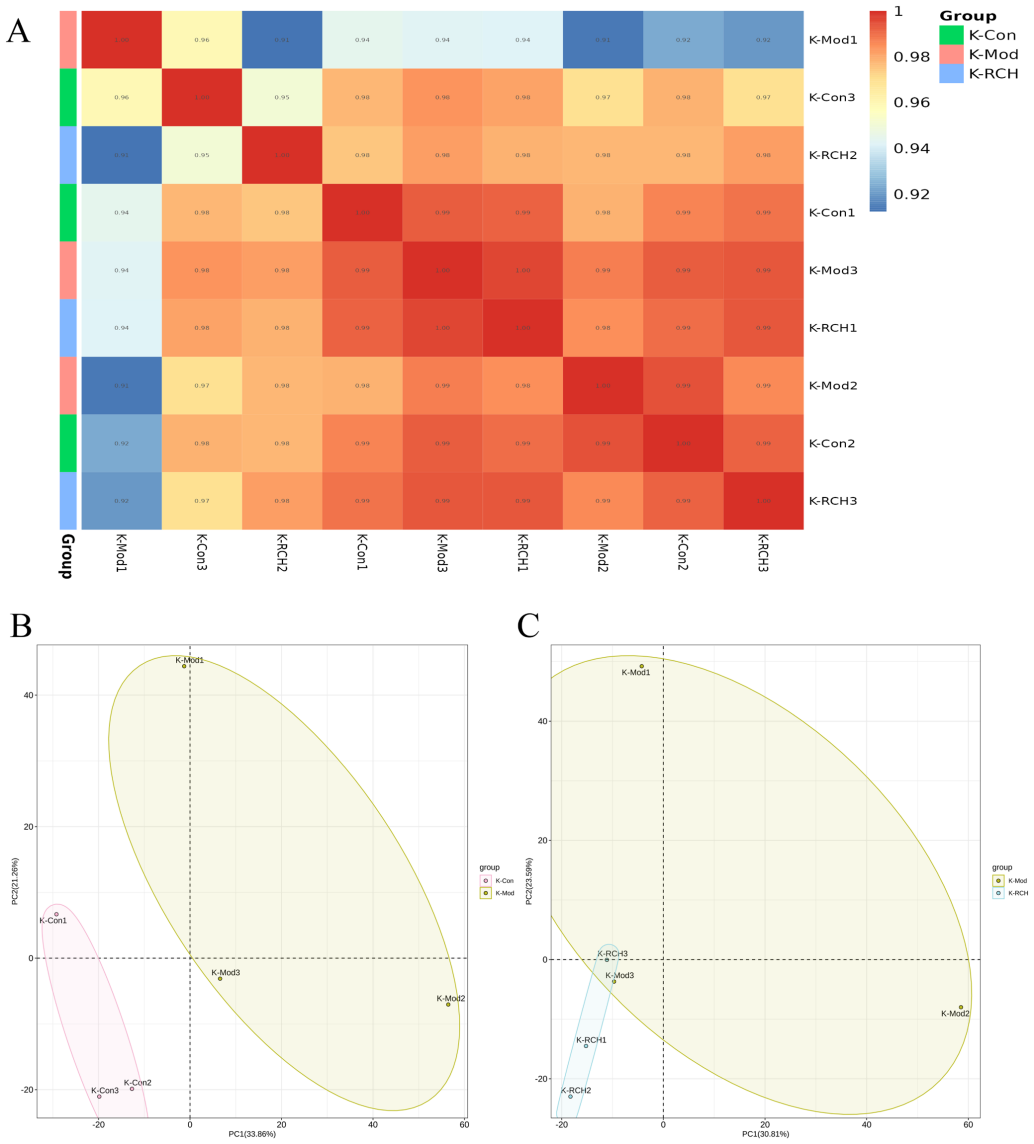
Transcriptome analysis of three groups of samples. (A) Pearson's correlation coefficient; (B) PCA in the control group and model group; (C) PCA in the model group and RCH group.

The correlation of gene expression level between samples reflects the similarity of expression level of samples with different treatments, which can be used to test the reliability of the experiment and the rationality of sample selection. Pearson's Correlation Coecient r is an evaluation index of correlation. The closer r is to 1, the stronger the correlation between the two samples is. Generally speaking, a correlation coefficient greater than 0.8 indicates a very strong good correlation (Rooney et al. 2023). In this study, the Pearson correlation coefficients between samples were all greater than 0.9, indicating a strong correlation between samples (Figure 5A). At the same time, PCA showed good separation among the three groups of data (Figure 5B,C).

**FIGURE 6:**
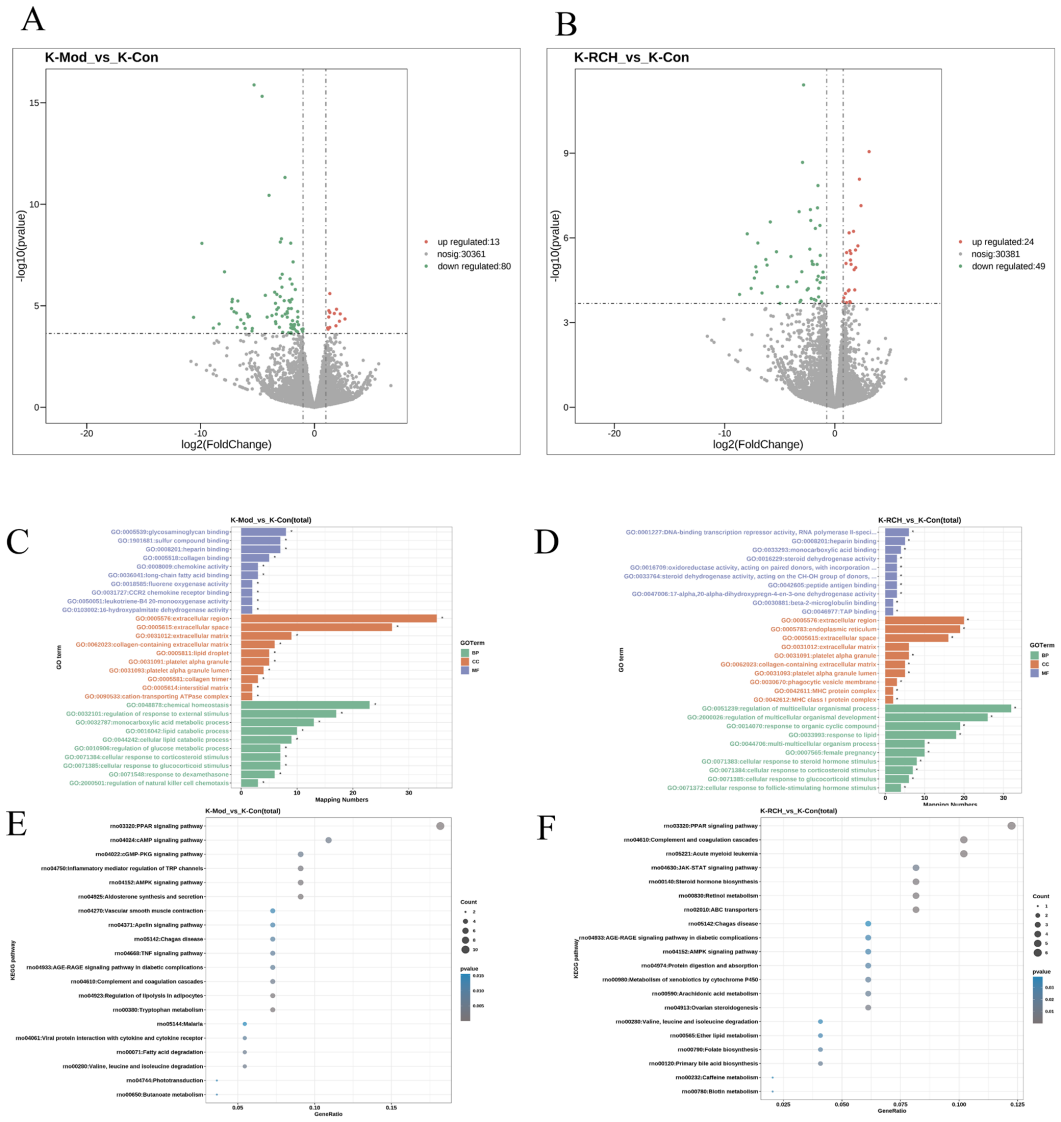
Differential genes and enrichment analysis. (A, B), (C, D), (E, F) are the differential genes between the control group and the model group, and the model group and the RCH group respectively, with GO enrichment and KEGG enrichment.

Compared with Con group, 93 differential genes were screened (of which 13 genes were significantly up‐regulated and 80 genes were significantly down‐regulated) from Mod group, indicating that there was a disorder in gene expression in the kidney during TGT administration (Figure 6A). In order to better understand the correlation between differential genes, GO and KEGG pathway enrichment analysis was performed on differential genes. In GO analysis, after TGT treatment, differentially expressed genes were significantly enriched in metabolic pathways such as lipid catabolic process, cellular lipid catabolic process, monocarboxylic acid metabolic process, regulation of glucose metabolic process, indicating that TGT has a disruptive effect on metabolism (Figure 6C). In KEGG enrichment analysis, the differential genes were mainly concentrated in Tryptophan metabolism, Valine, leucine and isoleucine degradation, Fatty acid degradation, AGE‐RAGE signaling pathway in diabetic complications, AMPK signaling pathway, cAMP signaling pathway, TNF signaling pathway and PPAR signaling pathway (Figure 6E). These pathways are closely related to glycolipid metabolism, inflammation and immune regulation. More importantly, the enrichment analysis further located regulatory links closely related to ferroptosis. DEGs were significantly enriched in entries related to response to metal ions (GO: 0010038) and oxidative stress response, suggesting that RCH may reduce oxidative damage by maintaining iron homeostasis in kidney cells, which is consistent with the results of ELISA.

After RCH intervention, the gene expression of kidney did not completely recover to the same state that of healthy kidney. By screening for differential genes, a total of 73 significantly changed genes were identified, of which 24 genes were significantly up‐regulated and 49 genes were significantly down‐regulated (Figure 6B). GO and KEGG enrichment analysis were carried out to explore the correlation between different genes. In GO analysis, the enrichment was concentrated in cellular response to corticosteroid stimulus, response to lipid, steroid dehydrogenase activity (Figure 6D). On this basis, further KEGG pathway enrichment analysis showed that in addition to being enriched in the PPAR signaling pathway, AMPK signaling pathway, AGE‐RAGE signaling pathway in diabetic complications, JAK–STAT and other inflammation, immunity, and cell apoptosis signaling pathways, DEGs were more intensively involved in the metabolism reprogramming process, including key metabolic pathways such as valine, leucine and isoleucine degradation, arachidonic acid metabolism and tryptophan metabolism (Figure 6F). Of particular importance, the KEGG results also specifically targeted the Peroxisome pathway, the core line of defense against ferroptosis, which is mutually corroborated with the findings on regulation of oxidoreductase activity in GO analysis, and is consistent with the expression trend of SOD and GSH‐Px in kidney tissue. All these indicate that RCH may improve energy substrate utilization by reshaping the “inflammation‐metabolism” network, and rely on the peroxisome system to reduce lipid peroxidation damage, thereby blocking the ferroptosis process of kidney cells and ultimately reversing the pathological state of kidney injury.

The results were consistent with the findings of network pharmacology. More and more evidence shows that the mechanisms of oxidative stress, inflammation, and apoptosis play a key role in the occurrence and progress of drug nephrotoxicity (Raza and Naureen 2020; Gao et al. 2021). The activation of advanced glycation end products (AGE) and its receptor RAGE is the key promoting factor of chronic inflammation. The increase of glycosylation reactions and oxidative stress leads to the accumulation of toxic compound AGE in the body. AGEs can activate NF‐κB and other signaling pathways by binding with RAGE, promote the release of inflammatory factors, and aggravate cell injury, inflammation, and apoptosis (Zhou et al. 2024; Bhattacharya et al. 2023). The JAK/STAT signaling pathway is increasingly involved in the pathophysiological process of kidney diseases. Experimental evidence shows that inhibiting the JAK/STAT signaling pathway, especially JAK2 and STAT3, leads to low expression of downstream inflammatory factors TNF‐α and IL‐6, which may inhibit kidney fibrosis and protect kidney function (Matsui and Meldrum 2012; Özmen et al. 2025). The study has pointed out that the inflammatory pathway driven by TNF‐α through TNF receptors 1 (TNFR1) and 2 (TNFR2) involves important mediators in the pathogenesis of chronic kidney disease (CKD) (Lousa et al. 2022). Clinical studies have found that TNF‐α can stimulate glomerular mesangial cells to synthesize endothelin‐1, which has a direct cytotoxic effect on kidney tissue, and increased cytokine production induces apoptosis and necrosis of glomerular cells (Ryabova and Rakityanskaya 2022). Therefore, interfering with the production of TNF‐α by inhibiting the activation of the TNF signaling pathway can inhibit TNF‐α‐induced cell death and NF‐κB activation, which may delay the progression of kidney injury (Al‐Lamki and Mayadas 2015).

### 3.5 Screening and Pathway Enrichment Analysis of Potential Differential Metabolites

**FIGURE 7:**
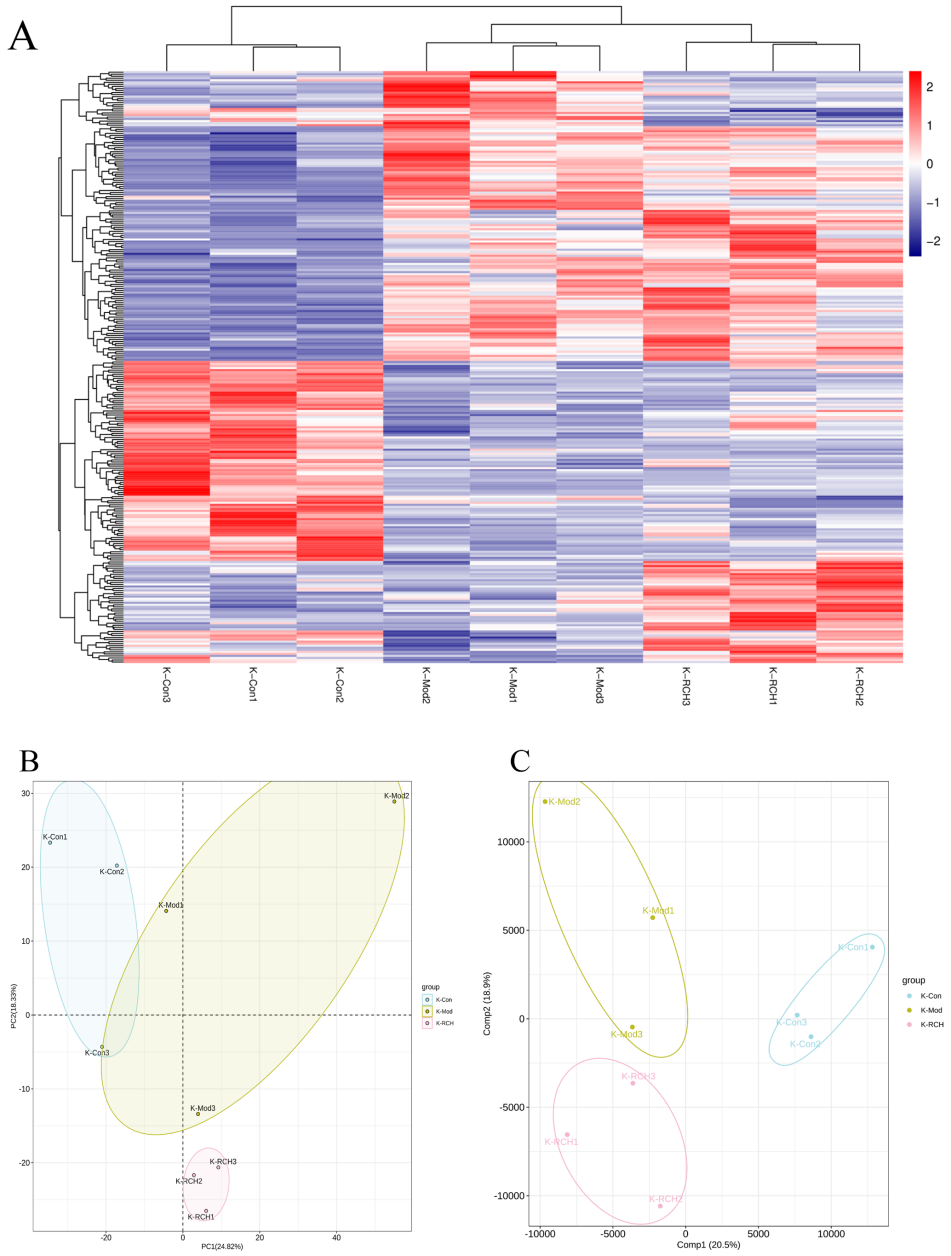
Identification and enrichment analysis of differential metabolites. (A) Metabolites heat map; (B) PCA map of three groups of samples; (C) OPLS‐DA map of three groups of samples.

Cluster heat map analysis showed significant differences in metabolites among the three groups (Figure 7A). Unsupervised PCA captured the characteristics of the principal components (Figure 7B). To identify differential metabolites in each group of kidneys, the supervised OPLS‐DA method was used to maximize inter‐group resolution. OPLS‐DA analysis showed that the three groups of samples were separated from each other and each internal group was well aggregated (Figure 7C). The R2Y and Q2 indicators were used to evaluate the quality of the OPLS‐DA model. In this study, the R2Y and Q2 values of the Con and Mod groups, and the Mod and RCH groups were 0.998, 0.677 and 0.999, 0.776 respectively, indicating that the OPLS‐DA model was highly reliable.

**FIGURE 8:**
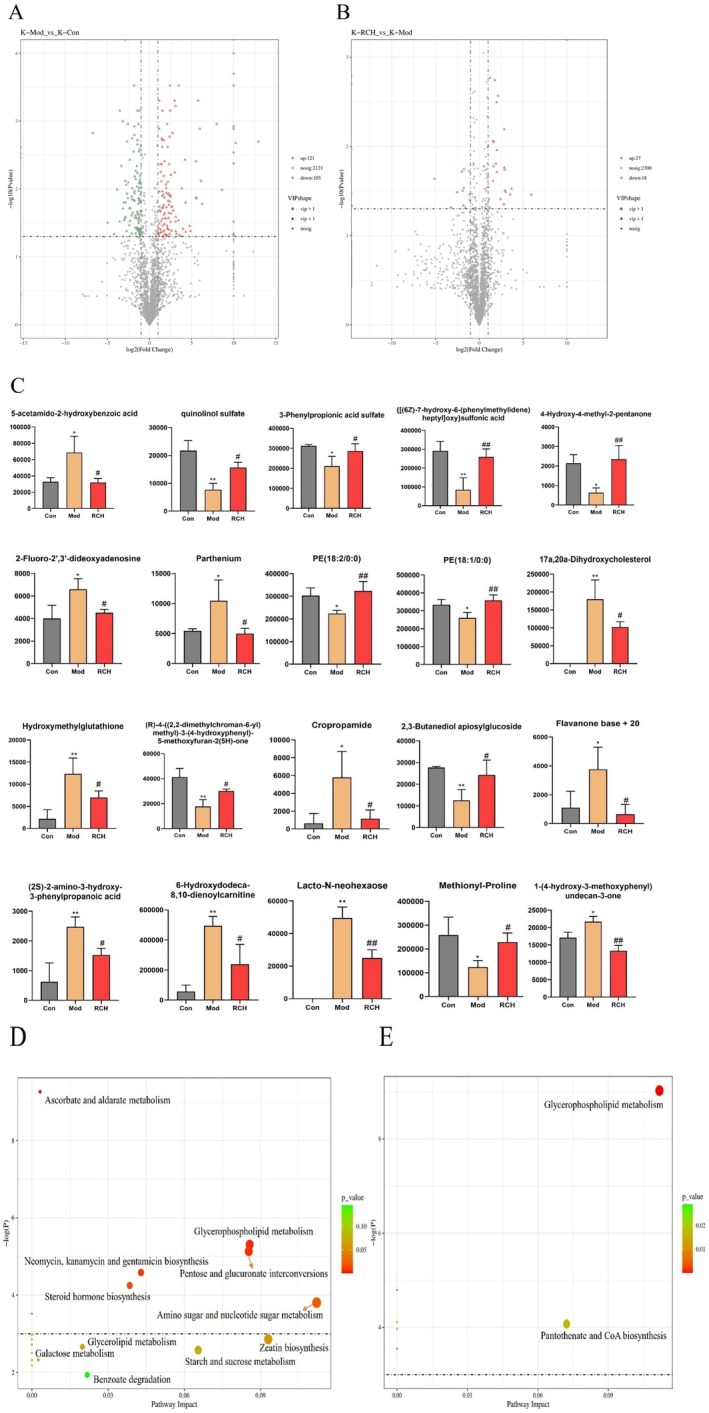
(A) Volcano plot between the control group and model group; (B) Volcano plot between the model group and RCH group; (C) 20 biomarkers; (D) Enrichment pathways of differential metabolites in the control group and model group; (E) Enrichment pathways of differential metabolites in the model group and RCH group. Compared with the control group, *p < 0.05 or **p < 0.01 Compared with the model (TGT) group, ^#^ *p* < 0.05 or ^##^ *p* < 0.01.

In order to further explore the kidney protective mechanism of RCH, we detected the changes of metabolites in rat kidney tissue induced by TGT after RCH intervention. The results showed that compared with the Con group, 121 metabolites were up‐regulated and 103 metabolites were down‐regulated in the Mod group (Figure 8A). Compared with the Mod group, 27 metabolites were significantly up‐regulated and 18 metabolites were significantly down‐regulated in the RCH group (Figure 8B). Subsequently, one‐factor analysis was performed on the Volcano Plot, and according to the determined FC values and the significance of the differences, we finally identified 20 metabolites as candidate biomarkers from rat kidney samples (Figure 8C and Table 3). Among them, biomarkers 17a, 20a‐dihydrocholestol, 5‐acetamido‐2‐hydroxybenzoic acid and hydroxymethyl glutathione in Mod group were significantly up‐regulated, while PE (18:1/0:0) and PE (18:2/0:0) were significantly down‐regulated. RCH intervention reduced the levels of 17a, 20a‐dihydrocholesol, 5‐acetamido‐2‐hydroxybenzoic acid and Hydroxymethylglutathione, and increased the levels of PE (18:1/0:0) and PE (18:2/0:0). 17a,20a‐Dihydroxycholesterol is an intermediate in C21‐Steroid hormone metabolism and the 8th to last step in the synthesis of Cortolone and is converted from 20alpha‐Hydroxycholesterol via the enzyme cytochrome P450 (EC1.14.99.9). It is then converted to 17alpha‐Hydroxypregnenolone via the enzyme cytochrome P450 (EC1.14.15.6), however, as an agonist of GPR56, 17 alpha‐hydroxypregnenolone can effectively resist iron death (Lin, Ma, et al. 2024). Cummins et al. (2011) pointed out that Hydroxymethylglutathione can detoxify the herbicide benzoxazole. 5‐acetamido‐2‐hydroxy Benzoic acid has anti‐inflammatory effect and can effectively reduce the edema formation caused by carrageenan oil (Santos et al. 2023). Phosphatidylethanolamine (PE) is one of the main components of the cell membrane and plays a key role in maintaining cell structure and function, which may determine the lipid metabolism and immune response of macrophages (Maurício et al. 2025). Therefore, in this study, TGT may stimulate the production of 17a,20a‐Dihydroxycholesterol, 5‐acetamido‐2‐hydroxybenzoic acid and Hydroxymethylglutathione, playing anti‐inflammatory and detoxifying effects. RCH intervention may promote the production of phospholipids such as PE (18:1/0:0) and PE (18:2/0:0), inhibit toxic reactions such as inflammation, and down‐regulate the levels of 17a, 20a‐dihydrocholestol, 5‐acetamido‐2‐hydroxybenzoic acid and Hydroxymethylglutathione.

**TABLE 3:** Identification results of differential metabolites in two comparisons.

No.	Metabolite	Formula	Ion mode	RT/Min	Measured m/z
1	5‐Acetamido‐2‐hydroxybenzoic acid	C_9_H_9_NO_4_	[M − H]−	3.43	194.0461
2	Quinolinol sulfate	C_9_H_7_NO_4_S	[M − H]−	3.69	270.0074
3	3‐Phenylpropionic acid sulfate	C_9_H_10_O_5_S	[M − H]−	3.93	275.0227
4	{[(6Z)‐7‐hydroxy‐6‐(phenylmethylidene)heptyl]oxy} sulfonic acid	C_14_H_20_O_5_S	[M − H]−	3.93	321.0764
5	4‐Hydroxy‐4‐methyl‐2‐pentanone	C_6_H_12_O_2_	[M − H]−	4.87	115.0764
6	2‐Fluoro‐2′,3′‐dideoxyadenosine	C_10_H_12_FN_5_O_2_	[M − H]−	4.90	234.0772
7	Parthenium	C_15_H_20_O_3_	[M − H]−	5.81	283.1102
8	PE (18:2/0:0)	C_23_H_44_NO_7_P	[M − H]−	6.35	476.2782
9	PE (18:1/0:0)	C_23_H_46_NO_7_P	[M − H]−	6.82	478.2937
10	17a,20a‐Dihydroxycholesterol	C_27_H_46_O_3_	[M + H]+	3.88	463.3176
11	Hydroxymethylglutathione	C_11_H_19_N_3_O_7_S	[M + H]+	4.00	355.1295
12	(R)‐4‐((2,2‐dimethylchroman‐6‐yl)methyl)‐3‐(4‐hydroxyphenyl)‐ 5‐methoxyfuran‐2(5H)‐one	C_23_H_24_O_5_	[M + H]+	4.17	422.1964
13	Cropropamide	C_13_H_24_N_2_O_2_	[M + H]+	4.23	498.4022
14	2,3‐Butanediol apiosylglucoside	C_15_H_28_O_11_	[M + H]+	4.30	448.1759
15	Flavanone base +2O	C_15_H_12_O_4_	[M + H]+	4.32	257.0814
16	(2S)‐2‐amino‐3‐hydroxy‐3‐phenylpropanoic acid	C_9_H_11_NO_3_	[M + H]+	4.34	164.0709
17	6‐Hydroxydodeca‐8,10‐dienoylcarnitine	C_19_H_33_NO_5_	[M + H]+	4.44	356.2436
18	Lacto‐N‐neohexaose	C_40_H_68_N_2_O_31_	[M + H]+	4.69	559.1767
19	Methionyl‐Proline	C_10_H_18_N_2_O_3_S	[M + H]+	4.70	247.1116
20	1‐(4‐hydroxy‐3‐methoxyphenyl)undecan‐3‐one	C_18_H_28_O_3_	[M + H]+	6.13	310.2382

MetPA enrichment analysis of differential metabolites was performed using the metabolomic database. The results showed that the differential metabolites in the Con and Mod groups were mainly enriched in Glycerophospholipid metabolism, ascorbate and aldarate metabolism, amino sugar and nucleotide sugar metabolism, and Starch and sucrose metabolism (Figure 8D). In the Mod and RCH groups, differential metabolites were significantly enriched in the pathways of Glycerophospholipid metabolism, Pantothenate and CoA biosynthesis (Figure 8E). Importantly, Glycerophospholipid metabolism is a co‐enrichment pathway. Signature metabolites 17a,20a‐Dihydroxycholesterol and Pantetheine were identified to be enriched in significantly enriched in Steroid hormone biosynthesis (C05499) and Pantothenate and CoA biosynthesis (C00831) pathways (*p* < 0.05, impact > 0), respectively. Nephrotoxicity caused by disorders of the glycerophospholipid metabolic pathway is often accompanied by significant changes in key metabolites, activating NF‐κB signaling transduction (Wang, Yang, et al. 2023), which is consistent with the mechanism information predicted by network pharmacology. The first step in the synthesis of glycerophospholipid is to synthesize phosphatidic acid, and then to synthesize PE, phosphatidylinositol (PI), phosphatidylserine, phosphatidylcholine, cardiolipin through diglyceride pathway and cytidine diphosphate diglyceride pathway. Then, the lysophospholipids generated by hydrolysis of phospholipase A1 or A2, such as lysophosphatidic acid, lysophosphatidylcholine and lysophosphatidylinositol, can play a molecular signal role and play an important role in the occurrence of kidney diseases (Vance 2015), which is consistent with the changes of landmark metabolites PE(18:1/0:0) and PE(18:2/0:0). However, the gene expression of RCH regulating TGT‐induced nephrotoxicity is still unclear, so further research is needed to clarify the mechanism behind the protective effect of RCH.

### 3.6 Regulation of RCH on Kidney Metabolic Pathway, Inflammation and Oxidative Stress

**FIGURE 9:**
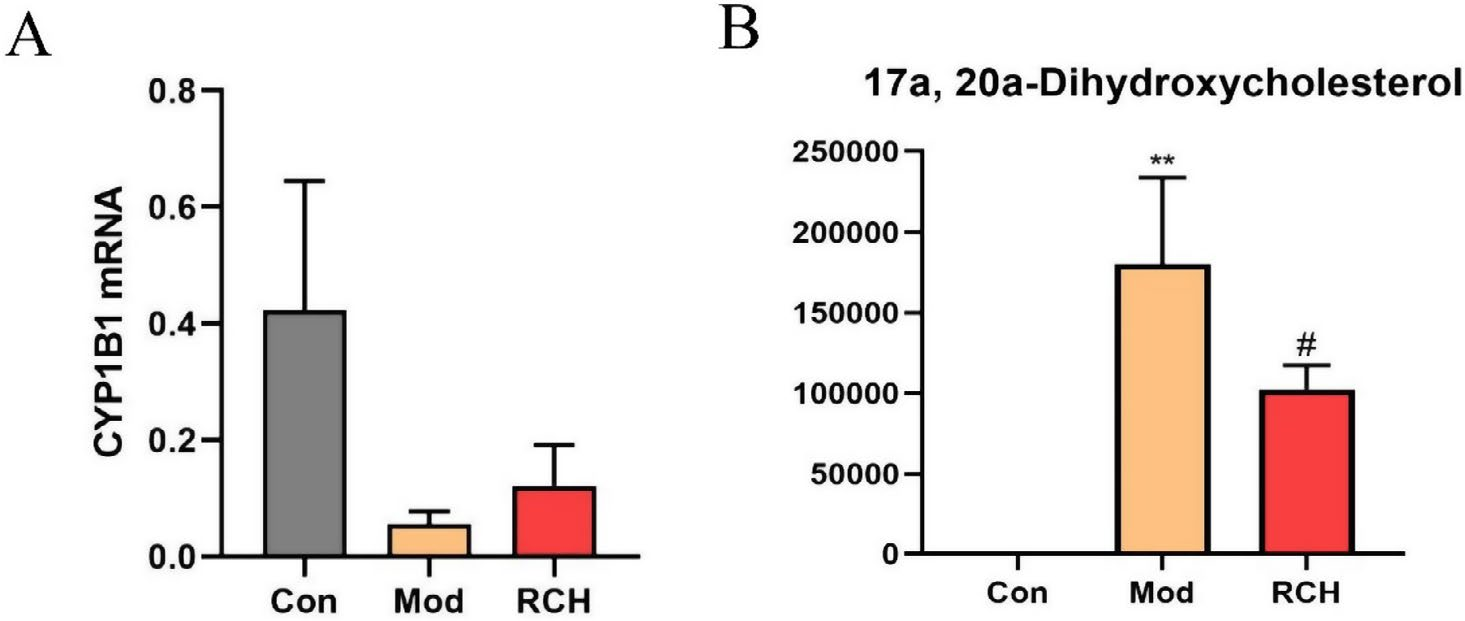
Changes of metabolite 17a,20a‐Dihydroxycholesterol (A) and CYP1B1 gene (B) in steroid hormone biosynthesis pathway. Compared with the control group, **p < 0.01; compared with the model (TGT) group, ^#^ *p* < 0.05.

In order to further explore the mechanism of RCH on the metabolic pathway of kidney damage, we analyzed the Steroid hormone biosynthesis. The results showed that TGT down‐regulated CYP1B1 gene, but RCH up‐regulated CYP1B1 gene (Figure 9A). The expression of metabolite 17a, 20a‐dihydrocholestol in the Mod group was lower than that in the Con group, while RCH intervention upregulated the expression of 17a, 20a‐Dihydroxycholesterol (Figure 9B).

**FIGURE 10:**
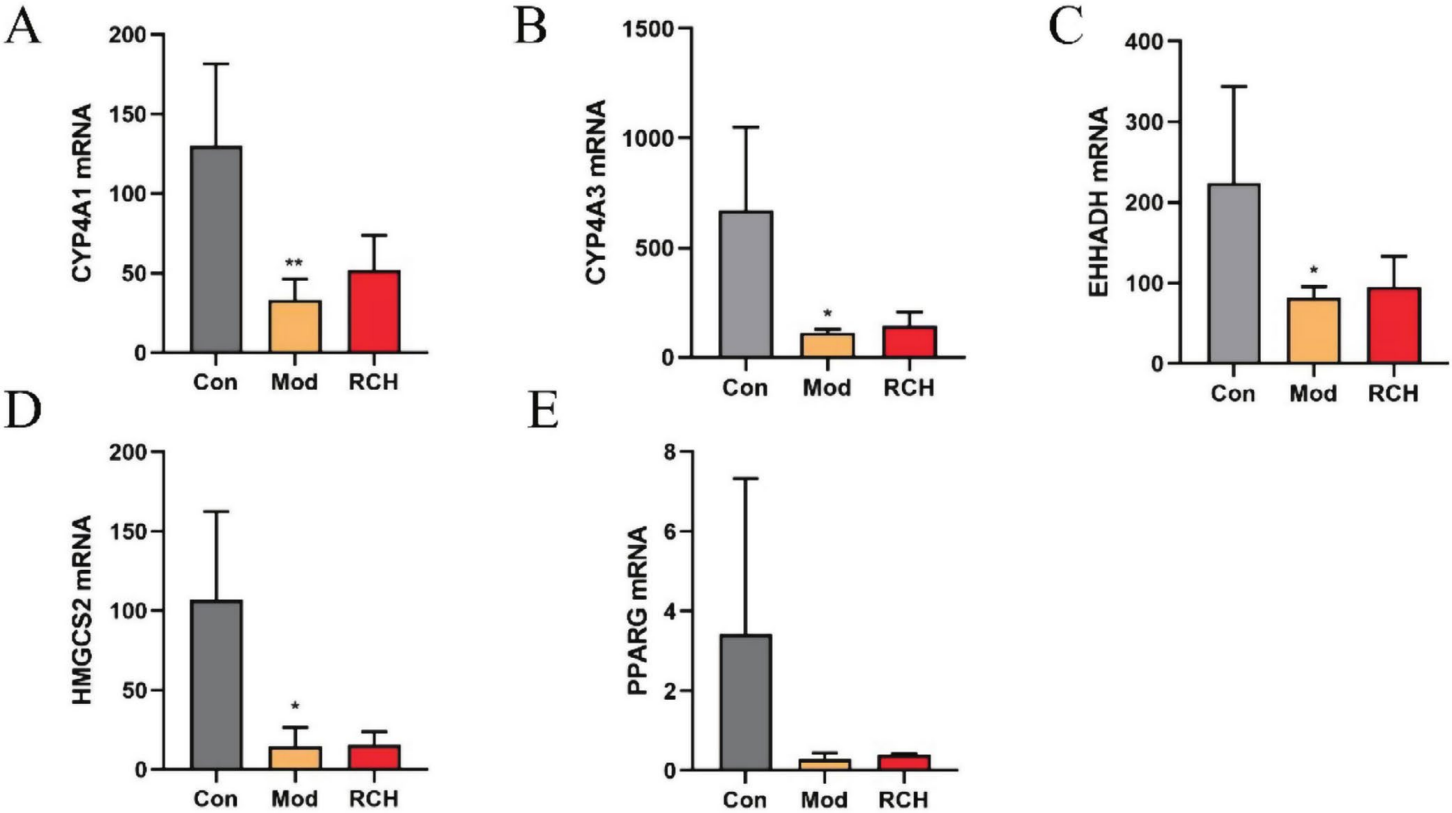
The expression levels of key genes. (A) CYP4A1 expression; (B) CYP4A3 expression; (C) EHHADH expression; (D) HMGCS2 expression; (E) PPARG expression. Compared with the control group, *p < 0.05 or **p < 0.01.

Compared with the Con group, the expression levels of several key genes CYP4A1, CYP4A3, EHHADH, HMGCS2, and PPARG belonging to the PPAR signaling pathway and Steroid hormone biosynthesis were down‐regulated in the Mod group, indicating that TGT had an inflammatory reaction in the kidney after long‐term high‐dose oral administration, while RCH intervention up‐regulated the expression of these genes (Figure 10).

**FIGURE 11:**
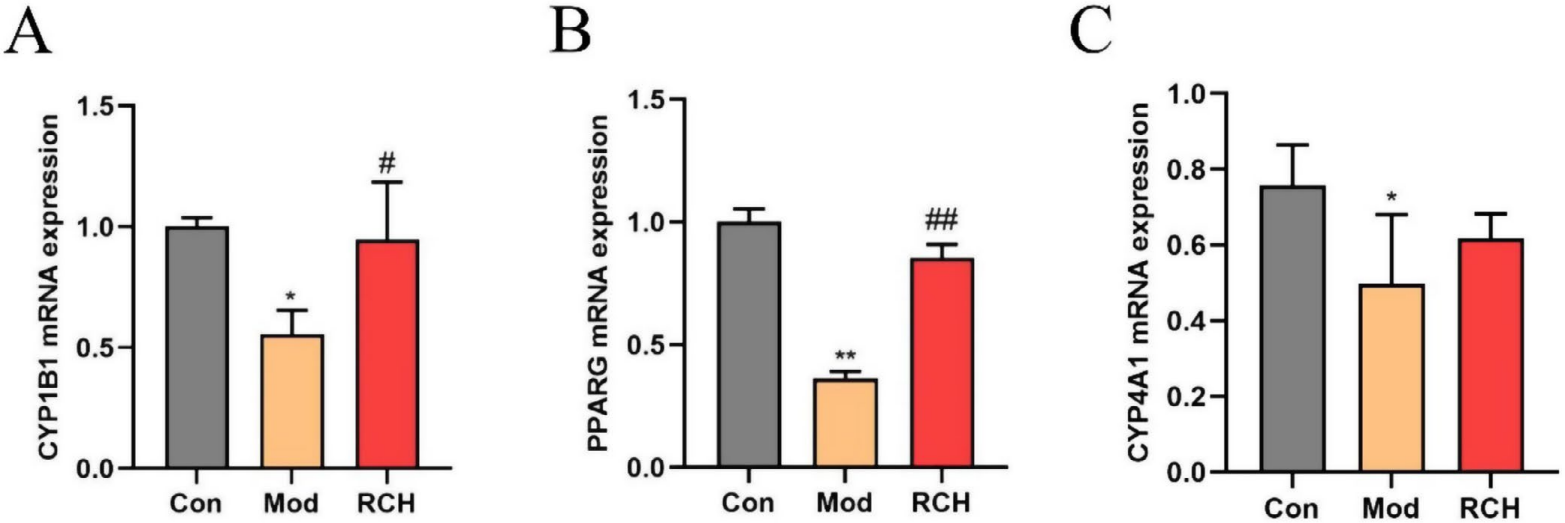
The results of qRT‐PCR. (A) CYP1B1 mRNA expression; (B) PPARG mRNA expression; (C) CYP4A1 mRNA expression. Compared with the control group, *p < 0.05 or **p < 0.01. Compared with the model (TGT) group, ^#^ *p* < 0.05 or ^##^ *p* < 0.01.

The results of qRT‐PCR (Figure 11) showed that the expressions of CYP1B1, CYP4A1, and PPARG mRNA in the model group were lower than those in the control group (*p* < 0.01, *p* < 0.05), while the expressions of CYP1B1 and PPARG mRNA in the RCH group were higher than those in the model group (*p* < 0.01, *p* < 0.05). Although the expression of CYP4A1 mRNA was also higher than that in the model group, the difference was not statistically significant (*p* > 0.05). These results are basically consistent with transcriptomic analysis.

CYP4A1 and CYP4A3 are biomarker genes related to drug toxicity (Hasan et al. 2019), and CYP1B1, as a key drug metabolic enzyme, can metabolize endogenous compounds and clinical drugs (Huang, Hu, et al. 2025). Previous study has shown that activation of CYP1B1 can counteract cisplatin‐induced nephrotoxicity (Franzin et al. 2023). They may be the key enzymes for the degradation of the biomarker metabolite 17a, 20a‐Dihydroxycholesterol to 17alpha‐Hydroxypregnolone. Therefore, the increase of 17a, 20a‐Dihydroxycholesterol level may be related to reduced expression of CYP4A1, CYP4A3, and CYP1B1, which slowed down their catabolic metabolism, while RCH changed this trend. In addition, the activation of cytochrome P450 enzyme may be synchronized with the up‐regulation of the expression of ABC transporters (abcc1, abcc2, and abcg2), so it can be speculated that RCH may reduce kidney damage by accelerating the excretion of toxicants (Yuan et al. 2014; Lemmen et al. 2013). Studies have found that the energy demand of renal proximal tubule cells depends on β‐oxidation of mitochondrial fatty acids, and EHHADH is a key enzyme for the oxidation of mitochondrial fatty acids (Tanaka et al. 2026). In cells with EHHADH gene knockdown, reactive oxygen species (ROS) level is increased and the renal tubular damage is aggravated (Kan et al. 2024). There is evidence that ketone bodies can play an antioxidant role. Mitochondrial 3‐hydroxy‐3‐methylglutaryl‐coenzyme A synthase (HMGCS2) in kidney is a gene that regulates the production of ketone bodies, and its up‐regulation can inhibit the phosphorylation of Akt, reduce the MDA level and SOD activity in the kidney tissue of Stroke‐Prone spontaneously Hypertensiverats (SHRSSPS) (Yi et al. 2010), and can also alleviate colon injury, oxidative stress and inflammation (Wang, Zhao, et al. 2023). Several studies have shown that PPARG plays a key role in glucose homeostasis and lipid metabolism (Wu et al. 2019). Lipid accumulation can trigger a lipotoxic reaction, which will aggravate kidney damage in patients with diabetic nephropathy (DKD). Activation of the PPARA/PPARG‐UCP1 pathway can significantly improve the oxidation efficiency of fatty acid, reduce ROS production, and effectively alleviate lipotoxic reactions (Yong et al. 2025). The test results of qRT‐PCR further show that RCH can alter inflammation and oxidative stress through its effects on key genes in Steroid hormone biosynthesis and PPAR signaling pathway, thereby improving kidney damage.

### 3.7 Integrated Analysis of Transcriptomics and Metabolomics

**FIGURE 12:**
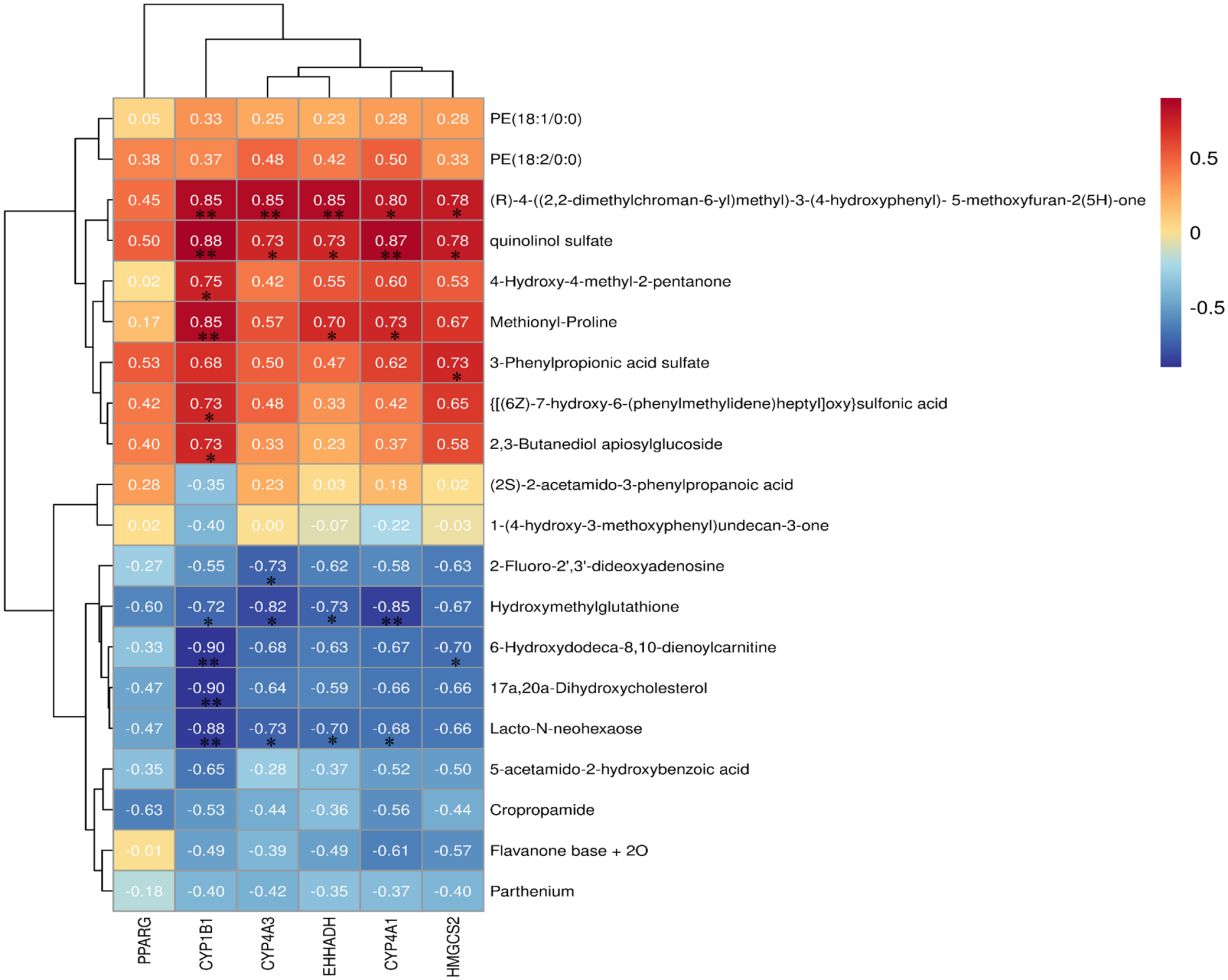
Correlation analysis of DEGs and metabolites. *p < 0.05, **p < 0.01.

The “gene‐metabolite” heat map (Figure 12) constructed through Spearman correlation analysis in the OmicStudio tool (Lyu et al. 2023) shows that there is a close co‐expression relationship between the 6 significantly regulated central genes screened by the transcriptome and 20 key molecules in the metabolome. In particular, CYP1B1 showed a significant negative correlation with 17a, 20a‐Dihydroxycholesterol, 6‐Hydroxydodeca‐8,10‐dienoylcarnitine, and Lacto‐*N*‐neohexaose (|*r*| > 0.8, *p* < 0.01), while it showed a significant positive correlation with quinoline sulfate and Methionyl−Proline (|*r*| > 0.8, *p* < 0.01). CYP4A3 showed a significant positive correlation with (R)‐4‐((2,2‐dimethylchroman‐6‐yl)methyl)‐3‐(4‐hydroxyphenyl)‐5‐methoxyfuran‐2(5H)‐one (|*r*| > 0.8, *p* < 0.01), and a significant negative correlation with hydroxymethylglutathione (|*r*| > 0.8, *p* < 0.05). CYP4A1 showed a significant positive correlation with quinolinol sulfate (|*r*| > 0.8, *p* < 0.01), and a significant negative correlation with Hydroxymethylglutathione (|*r*| > 0.8, *p* < 0.01). EHHADH showed a significant positive correlation with (R)‐4‐((2,2‐dimethylchroman‐6‐yl)methyl)‐3‐(4‐hydroxyphenyl)‐5‐methoxyfuran‐2(5H)‐one (|*r*| > 0.8, *p* < 0.01). There was a significant positive correlation mainly among HMGCS2 and (R)‐4‐((2,2‐dimethylchroman‐6‐yl)methyl)‐3‐(4‐hydroxyphenyl)‐5‐methoxyfuran‐2(5H)‐one, quinolinol sulfate and 3‐Phenylpropionic acid sulfate (|*r*| > 0.7, *p* < 0.05), and a significant negative correlation with 6‐Hydroxydodeca‐8,10‐dienoylcarnitine (|*r*| = 0.7, *p* < 0.05). PPARG only showed a moderate positive correlation with 3‐Phenylpropionic acid sulfate (|*r*| > 0.5), and a moderate negative correlation with Hydroxymethylglutathione and Cropropamide (|*r*| > 0.5). These results suggest that, similar to TNF‐a, Cleavedcaspase‐3 and SIRT1 proteins, these genes may be the core pharmacological targets of RCH in regulating the renal microenvironment. However, although the regulation of CYP1B1, CYP4A1, and PPARG genes has been verified, the linkage regulation mechanism with metabolites will be focused on verification in subsequent experiments.

## 4 Conclusion

This study employed network pharmacology, transcriptomics, and metabolomics to investigate RCH's protective effects against TGT‐induced nephrotoxicity. Studies have found that RCH regulates the metabolism of 17a,20a‐dihydroxycholesterol and may act through steroid hormone biosynthesis and glycerophospholipid metabolism pathways. RCH also regulates the expression of cytochrome P450 enzymes and PPAR pathway‐related genes, and significantly reduces kidney damage by reducing inflammation, reducing oxidative stress, and enhancing detoxification. These findings highlight RCH's potential for treating nephrotoxicity and suggest new therapies addressing TGT's clinical limitations. Future research should explore species differences in metabolites and the exact links between metabolites, genes, and pathways.

## Author Contributions

**Tong Zhou:** visualization, formal analysis, data curation. **Cong Zhang:** writing – original draft, validation, methodology, funding acquisition, formal analysis, conceptualization. **Zepeng Zhang:** validation, supervision, project administration, writing – review and editing. **Lei Zhang:** writing – review and editing, validation, supervision, project administration, funding acquisition, conceptualization.

## Funding

This work was supported by Suzhou Pharmaceutical Association‐Jiangsu Hengrui Medical Clinical Pharmacy Research Fund (Syhky202308), Jiangsu Association of Chinese Medicine Research Fund‐Eaglet Soaring Project (CYTF2024047), Kunshan Key Research and Development Program (Social Development) (KS2541).

## Conflicts of Interest

The authors declare no conflicts of interest.

## Supporting information


**Table S1:** Primer sequences.

## Data Availability

The data that support the findings of this study are available on request from the corresponding author. The data are not publicly available due to privacy or ethical restrictions.
