# Supplementary material for: Network Pharmacology Combined With Metabonomics and Transcriptomics Reveals the Mechanism of Rubus Chingii Hu in Alleviating Nephrotoxicity Caused by Tripterygium Glycosides Tablet
Source: Food Sci Nutr. 2026 Aug 4;14(8):e72189. doi: 10.1002/fsn3.72189 (PMC13434843; doi:10.1002/fsn3.72189)
Supplement: Supplementary file 1 [file FSN3-14-e72189-s001.docx]

**TABLE S1**  Primer sequences

| Gene | Direction | Primer sequence |
| --- | --- | --- |
| CYP4A1 | Forward： | CACTGCCAACACCTCTGTTCTT |
|  | Reverse： | CAAGGA GCTCCATGGACTCT |
| CYP1B1 | Forward： | CTCTCTGGA GAA ATG GCCGA |
|  | Reverse： | AAAGGAAATCAAGCGCCACC |
| PPARG | Forward： | TCAAGAAGACGGAGACAGACA |
|  | Reverse： | TGGAAGAAGGGAAATGTTGG |
| β-actin | Forward： | TCGTGCGTGACATTAAAGAG |
|  | Reverse： | ATTGCCGATAGTGATGACCT |
